# Rapidly progressing periodontology

**DOI:** 10.4103/0972-124X.65425

**Published:** 2010

**Authors:** C. D. Dwarakanath

**Affiliations:** President, Department of Periodontology, Director of Postgraduate Studies, Oxford Dental College and Hospital, Hosur Road, Bangalore - 560 068, India. E-mail: drdwarakcd@yahoo.co.in

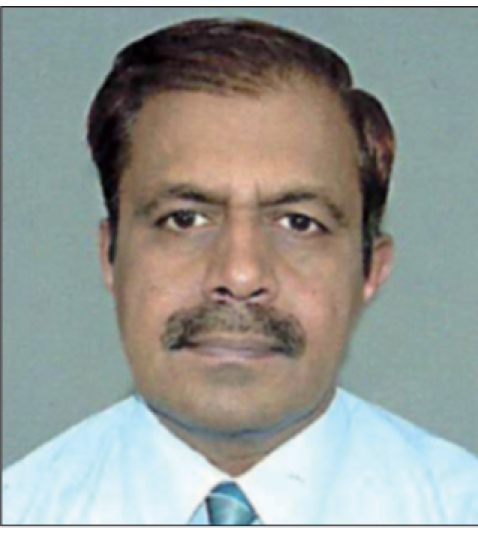



Periodontology as a specialty has witnessed immense progress over the last two decades. The scope and ambit of our specialty has expanded considerably with a lot of emphasis on microbiology, immunology, tissue engineering and the increasingly discussed periodontal medicine. Further, the surgical aspects have seen tremendous advances both in the techniques and equipments and materials. The pace with which knowledge has expanded is mind-boggling. All these factors have increased our responsibilities towards the profession. We have to keep pace with this rapid development in our field. Students have to work hard, spend more time getting information, whereas it is the duty of the teachers to guide them in the right direction. Our postgraduate curriculum provides ample opportunity to acquire knowledge. The journals and the internet are readily available sources of information. One has to utilize them to the maximum. The conferences, Continuing Dental Education (CDE) programs, workshops, etc., provide further platforms to learn. My earnest plea to all my young student friends is to take advantage of every available source to learn. Let there be no lack of zeal, enthusiasm and hunger in you to acquire knowledge. Merely adding M.D.S. to your names and becoming “flap specialists” will not take you anywhere. Your position and esteem in the medical profession deserve much more than what you are holding at present.

Wishing you all the best

